# Gastroblastoma in Adulthood—A Rarity among Rare Cancers—A Case Report and Review of the Literature

**DOI:** 10.1155/2019/4084196

**Published:** 2019-11-28

**Authors:** Giovanni Centonze, Alessandro Mangogna, Tiziana Salviato, Beatrice Belmonte, Laura Cattaneo, Melissa Anna Teresa Monica, Giovanna Garzone, Cecilia Brambilla, Alessio Pellegrinelli, Flavia Melotti, Adele Testi, Valentina Monti, Ketevani Kankava, Patrizia Gasparini, Gianpaolo Dagrada, Vincenzo Mazzaferro, Christian Cotsoglou, Paola Collini, Giancarlo Pruneri, Massimo Milione

**Affiliations:** ^1^Clinical Research Lab, Department of Pathology and Laboratory Medicine, Fondazione IRCCS – Istituto Nazionale dei Tumori, Milan, Italy; ^2^Tumor Genomics Unit, Department of Research, Fondazione IRCCS Istituto Nazionale dei Tumori, Milan, Italy; ^3^Clinical Department of Medical, Surgical and Health Science, University of Trieste, Ospedale di Cattinara, Trieste, Italy; ^4^Department of Diagnostic, Clinic and Public Health Medicine, University of Modena and Reggio Emilia, Modena, Italy; ^5^Tumor Immunology Unit, Department of Health Sciences, Human Pathology Section, University of Palermo, Palermo, Italy; ^6^First Pathology Division, Department of Pathology and Laboratory Medicine, Fondazione IRCCS – Istituto Nazionale dei Tumori, Milan, Italy; ^7^Department of Histopathology, Royal Brompton Hospital and Harefield NHS Trust, London, UK; ^8^Department of Pathology, ASST Franciacorta, Mellino Mellini Hospital, Chiari, Brescia, Italy; ^9^Servizio di Anatomia Patologica, Azienda Socio-Sanitaria Territoriale del Garda Presidio di Desenzano, Desenzano del Garda, Brescia, Italy; ^10^Second Pathology Division, Department of Pathology and Laboratory Medicine, Fondazione IRCCS – Istituto Nazionale dei Tumori, Milan, Italy; ^11^Teaching, Scientific and Diagnostic Pathology Laboratory, Tbilisi State Medical University, Tbilisi, Georgia; ^12^Hepato-Bilio-Pancreatic Surgery and Liver Transplantation, Fondazione IRCCS – Istituto Nazionale dei Tumori, Milan, Italy; ^13^School of Medicine, University of Milan, Milan, Italy

## Abstract

Gastroblastoma (GB) is a rare gastric epithelial-mesenchymal neoplasm, first described by Miettinen et al. So far, all reported cases described the tumor in children or young adults, and similarities with other childhood blastomas have been postulated. We report a case of GB in a 43-year-old patient with long follow up and no recurrence up to 100 months after surgery. So far, this is the second case of GB occurring in the adult age >40-year-old. Hence, GB should be considered in the differential diagnosis of microscopically comparable conditions in adults carrying a worse prognosis and different clinical approach.

## 1. Introduction

Gastroblastoma (GB) is a rare epithelial-mesenchymal gastric tumor featuring monotonous spindle and epithelial cells in relatively young patients [1]. It was first described by Miettinen et al. [1] as a biphasic epithelial-mesenchymal tumor of the stomach for which they proposed the term GB considering the similarity with the infantile blastoma and the analogy with other biphasic neoplasms of childhood where the term blastoma is used. Subsequently, other authors described similar biphasic gastric tumor in children and young adults and, only recently, Pinto et al. [2] observed a case of GB in the adult age. Therefore, to date, only ten case reports describe and illustrate GB among which only one occurred in adulthood [2, 3]. The tumor pathogenesis and biological potential is still unknown, and treatment remains a debatable issue [3].

Here, we report the second case of a GB in a >40 years old patient with clinical and follow up information, along with a review of the relative literature.

## 2. Case Description

A 43-year-old woman with unremarkable history was referred to our Institution following a generic diagnosis of a gastric tumor in another hospital center. In Following an intestinal bleeding, in September 2010, an endoscopic examination revealed a 2.5 cm submucosal, ulcerated lesion of the stomach, yet a first biopsy was not diagnostic material. The endoscopic ultrasound and a computed tomography (CT) scan confirmed the presence of an antral mass of 5 cm, originating from the muscularis propria with an endoluminal growth and a dishomogeneous enhancement. After two months, distal gastrectomy with a complete tumor resection was performed by means of laparoscopy.

Macroscopically, the resected antrum showed a transmural submucosal mass, mostly solid with a hemorrhagic cystic portion, measuring 5.3 cm in largest dimension with a grey cut surface. The overlying antral mucosa was normal and focally ulcerated. A microscopic evaluation revealed tumor involvement and was confined in the muscolaris propria of the gastric antrum.

**Figure 1:**
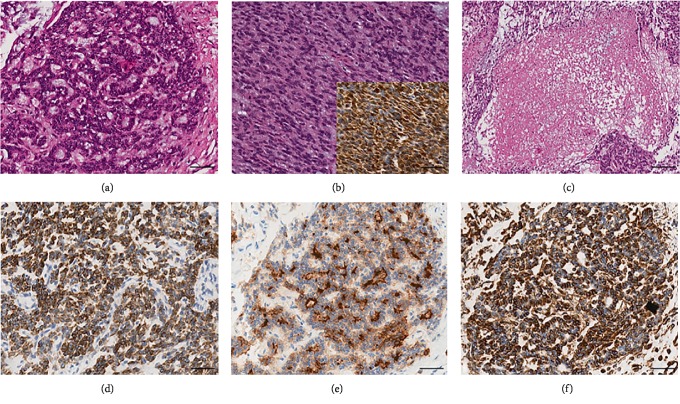
Gastroblastoma is a biphasic epithelial and mesenchymal tumor. Epithelial cells were characterized by round uniform nuclei, slightly eosinophilic cytoplasm, and inconspicuous nucleoli, are arranged also in glands or rosette-like structures containing luminal eosinophilic secretory material (a) and they showed strong pan-cytokeratin staining (d). Mesenchymal areas are organized in spindle cell fascicles (b) showing clear staining for vimentin (insert b). Necrosis is well represented (c). According to the biphasic nature of this neoplasm vimentin and CD10 are also expressed in epithelial glandular component (e–f). (Magnification 200x, scale bars 50 *µ*m.)

Histologically, the tumor showed a distinct biphasic pattern featuring epithelial areas haphazardly mixed with predominant spindle cell fascicles without any well-defined or abrupt transition (Figure 1). The epithelial component comprised epithelial cells displaying round uniform nuclei, a slightly eosinophilic cytoplasm, and inconspicuous nucleoli, mainly arranged in sheets, nests, cords and tubules (Figure 1(a)). Gland- or rosette-like structures showing dark and elongated nuclei were also present focally: luminal eosinophilic, secretory material was recognized as well (Figure 1(a)). On the other hand, the mesenchymal-type component was arranged in short fascicles or in a reticular pattern in loose stroma (Figure 1(b)). These cells possessed bland, oval to short spindle-shaped nuclei with inconspicuous nucleoli and scant cytoplasm (Figure 1(b)). Necrosis was well represented (Figure 1(c)). Mitoses were rare in both components. Two mitoses per 20 high-power fields (HPF) and zero mitoses per 20 HPF were observed in the mesenchymal and epithelial components, respectively. No evidence of lymphovascular/perineural tumor invasion was detected. Moreover, there were no lymph node metastases.

**Figure 2:**
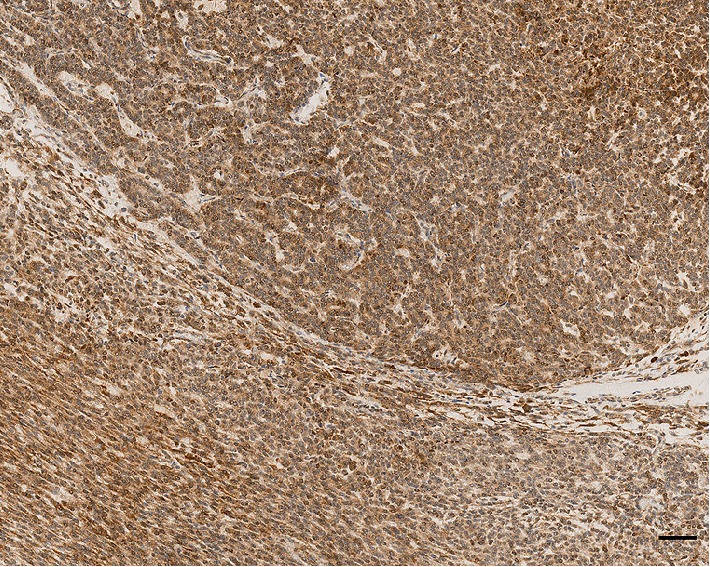
Both epithelial and spindle cell components displayed a strong and extensive positivity for GLI1 by immunohistochemistry in a nucleus as well as in the cytoplasm. (Magnification 100x, scale bar 50 *µ*m.)

As far as immunohistochemistry, the epithelial component mainly expressed pan-cytokeratin (Figure 1(d)), low-molecular-weight cytokeratin (LMWK), epithelial membrane antigen (EMA), CK 7 and CK 19 (but only focally). On the other hand, the spindle cell component was reported positive for vimentin (Figure 1(b)), while expression of CD10 was observed with a focal pattern. Both epithelial and spindle cell components displayed a strong and extensive positivity for GLI1 in a nucleus as well as in the cytoplasm (Figure 2). According to the biphasic nature of this peculiar malignancy vimentin and CD10 were also observed expressed in epithelial glandular component (Figure 1(e)–1(f)). No reactivity, however, was identified for c-KIT (CD117), DOG1, TLE1, CD34, CD99, inhibin, smooth muscle actin (SMA), CK 20, CK 5/6, CDX-2, S100, p63, TTF1, calretinin, synaptophysin, chromogranin, PDGFR-alfa, p16, estrogen and progesteron receptor (Table 1). Molecular cytogenetic characterization of *t*(X; 18) translocation, chromosomal rearrangement specific for synovial sarcoma, was investigated with fluorescent in situ hybridization (FISH) utilizing a commercial SS18 (SYT) probe (LSI SYT, Dual color, Break Apart Rearrangement Probe VYSIS). FISH analysis did not reveal SYT rearrangement, excluding the diagnosis of synovial sarcoma.

The patient was discharged after 11 days without any post-surgery complications and was included in an oncologic follow-up. Notably, at 100 months after surgery, the patient presents with no evidence of tumor recurrence or metastatic disease.

## 3. Discussion

GB is an extremely rare biphasic neoplasm firstly reported by Miettinen et al. in 2009 [1]. Since then, only nine reported cases occurred in pediatric age or young adulthood (<30 years) [1, 3–8]. Patients' age ranged from 9–30 years, with a mean of 22.6 years and it was slightly more frequent in men (Table 2). To date, no epidemiological studies reported incidence and prevalence of this malignancy, possibly due to the limited number of cases reported. Here, we describe a case report of a GB of the gastric antrum in a 43-year-old woman, with a particularly long follow up of 100 months with no recurrence after surgery. This is the second GB case described in an adult patient >40-year-old suggesting that this type of neoplasm it is not age-related. Due to the rarity of this disease, the etiopathogenesis of this tumor is unclear, and it is believed to develop from a multipotent stem cell [9]. Likewise, the malignant potential and appropriate treatment for GB remains disputable [3].

In most reported cases, GB patients present non-specific symptoms or even no symptoms at all and it is often accidentally found. Histologically, GB tumors are characterized by two components, the epithelial and the mesenchymal one, represented in variable portion, both with low-grade features, large tumor size, relatively low-mitotic activity, low overall atypia, absence of conspicuous nuclear pleomorphism and low malignant potential. Normally, the disease is delimited to the stomach, without metastatic potential or disease recurrence after curative resection [1, 2, 4–6, 8]. Nonetheless, two young adult patients, respectively 28 and 29 years, reported lymph node involvement and distant metastases [3, 7]. In 2012 Wey et al. [7] described a GB in a 28-year-old man with the similar biphasic architecture, bland cytology and the same histological and immunohistochemical features, but with microscopic evidence of regional lymph node metastasis. CT scan revealed clinical evidence of distant metastases to the liver and pelvis. Similarly, Toumi et al. [3] reported presence of regional nodal and distant metastases in a 29-year-old woman. Our case also shares a series of cytological, morphological and immunomorphological features with other reported cases including absence of metastasis and no recurrence 100 months after surgery (Tables 1 and 2).

The differential diagnosis for GB is challenging, including a number of biphasic malignant tumors. Particularly for adults, the combined morphologic, immunophenotypic and molecular features of our GB case allowed confident distinction from carcinosarcoma, inflammatory myofibroblastic tumor (IMT), teratoma, GIST, leiomyosarcoma, neuroendocrine tumors, mesothelial biphasic neoplasm and synovial sarcoma. Briefly, the most common biphasic tumor of the stomach that occurs in older patients is carcinosarcoma which shows, however, highly atypical squamous, adenocarcinomatous, or undifferentiated epithelial elements [10], resulting in poor clinical course with fatal outcome within a short period. In our case, no prominent inflammatory infiltrate similar to IMT and no cell differentiation spectrum characteristic of immature teratoma, as neural, neuroepithelial, cystic, epithelial, and cartilaginous component, were observed [11, 12]. GB differs from other neoplasms such as GIST, leiomyosarcoma, neuroendocrine tumors, mesothelial biphasic neoplasm and synovial sarcoma for its unique biphasic aspect and negativity for c-KIT, DOG1, SMA, desmin, CgA, SYN, calretinin, CK 5/6, TLE1 and SS18 (SYT) gene rearrangement [1, 13–17].

Recently, Graham et al. confirms the existence of GBs as a distinct entity, and demonstrate that they represent translocation-associated tumors, characterized by the presence of a somatic, recurrent, oncogenic MALAT1–GLI1 fusion gene [18]. The presence of this fusion gene causes the over-expression of GLI1 protein (Figure 2) and of several of its downstream targets with key roles in tumorigenesis [18, 19].

## 4. Conclusions

We reported a new case of GB occurring in an adult patient with a long follow-up. For this particular case, a conservative surgery was the curative treatment. Also based on the relative literature, it seems that this peculiar neoplasm pursues a favorable clinical course despite the adopted suffix “-blastoma”. Overall, it is crucial to report, describe and discuss each GB case presented to have a broader vision of the tumor from a pathological and morphological point of view. Hence, we believe that a prompt identification of GBs is important in clinical practice because it has a favorable prognosis if correctly managed.

## Figures and Tables

**Table 1:** Immunohistochemical profile of the different cases of gastroblastomas published in the literature.

	Miettinen et al. [1]		Shin et al. [8]		Wey et al. [7]		Yangyang Ma et al. [5]		Fernandes et al. [6]		Toumi et al. [3]		Pinto et al. [2]		Our case	
	E	S	E	S	E	S	E	S	E	S	E	S	E	S	E	S
SMA	−	−	−	−		−	−	−	−	−					−	−
Calretinin	−	−	−	−		−	−	−	−	−					−	−
CgA	−	−	−	−			−	−	−	−	−	−			−	−
NSE	−	−	−	−			−	−								
CD10	−	+	−	+ focal	+	+	+	+	+	+	+	+	+ focal	+	−	+ focal
CD34	−	−	−	−			−	−	−	−	−	−		−	−	−
CD56			+	+	+	+ focal	−	+	+	+	−	−	+ focal	+		
CD99	−	−					−	−			+	+			−	−
CDX2	−	−			−		−	−							−	−
Desmin	−	−	−	−		−	−	−	−	−				−		
DOG1	−	−					−	−	−	−			−		−	−
EMA	−		+	−		−	−	−			−	−			+ focal	
ER	−	−													−	−
PR															−	−
AE1/AE3	+	−	+	−	+	−	+	−	+	−			+	−	+	
CAM 5.2	+		+	−	+		+	−					+	−	+	
CK 5/6	−		−	−			+	−							−	
CK 7	+ focal	−			+ focal		−	−							+ focal	
CK 20	−	−			−		−	−			−	−			−	−
Inhibin			−	−	−	−	−	−							−	−
c-KIT (CD117)	−	−	+	−	+	−	−	−	−	−	−	−	−	−	−	−
p63	−	−	−	−	−		−	−							−	−
SYN	−	−	−	−			−	−	−	−				−	−	−
S100	−	−				−	−	−	−	−	−	−			−	−
TTF1	−	−													−	−
Vimentin	−	+	−	+	−	+	−	+	−	+	−	+		+	−	+
TLE1															−	−
GLI1															+	+

E: Epithelial component; S: Stromal component.

**Table 2:** Clinical characteristics of gastroblastomas reported in the literature.

Case	Age (yr)	Sex	Clinical features	Location	Tumor size (cm)	Lymph nodal/Distant metastases	Treatment	Follow-up (months)	Outcome
Miettinen et al. [1]	30	Male	Anemia, fatigue and abdominal mass	Gastric antrum	15 × 12	Absent	Antrectomy followed by radiation therapy	168	No recurrence
Miettinen et al. [1]	27	Female	Abdominal pain and mass	Greater curvature, gastric body	6 × 4 × 3.5	Absent	Partial gastrectomy	60	No recurrence
Miettinen et al. [1]	19	Male	Abdominal pain and mass	Greater curvature, gastric body	5 × 4 × 2.5	Absent	Subtotal gastrectomy	36	No recurrence
Pinto et al. [2]	53	Female	Heartburn and dyspepsia	Greater curvature, gastric antrum	2.3	Absent	Partial gastrectomy	18	No recurrence
Toumi et al. [3]	29	Female	Epigastric pain and hematemesis	Greater curvature, gastric body	7	Present	Partial gastrectomy with splenectomy	6	Recurrence
Na Zheng et al. [4]	12	Male	Bloody stool		7	Absent	Subtotal gastrectomy	8	No recurrence
Yangyang Ma et al. [5]	12	Male	Intermittent blood in stool and abdominal pain	Gastric antrum	4.5 × 2.5 × 2.5	Absent	Subtotal gastrectomy	8	No recurrence
Teresa Fernandez et al. [6]	19	Female	Abdominal pain and mass	Gastric antrum	10.5	Absent	Partial distal gastrectomy with lymphadenectomy	20	No recurrence
Wey et al. [7]	28	Male	Constipation and abdominal mass	Distal stomach	3.8 × 3.3 × 2.5	Present	Neoadjuvant chemotherapy followed by partial gastrectomy	3	Clinically stable. No new lesions
Shin et al. [8]	9	Male	Abdominal pain and mass	Gastric antrum	9 × 6.5	Absent	Distal gastrectomy	9	No recurrence
Our case	43	Female	Intestinal bleeding	Gastric antrum	5.3	Absent	Partial gastrectomy	100	No recurrence
